# Vascular access in out-of-hospital cardiac arrest with non-shockable rhythms—a retrospective observational study of the intravenous versus intraosseous approach: the VANISH study

**DOI:** 10.3389/fpubh.2026.1835203

**Published:** 2026-06-05

**Authors:** Amaya Burgos-Esteban, Manuel Quintana-Diaz, Valvanera Cordón-Hurtado, Marta Giménez-Luzuriaga, Marcos Juanes-García, Antonio Rodriguez-Calvo, Pablo Lasa-Berasain, Pilar Sanchez-Conde, Michał Czapla, Noelia Navas-Echazarreta, Ramón Baeza-Trinidad, Raúl Juárez-Vela

**Affiliations:** 1 Doctoral Program in Medicine and Surgery, University Autonomous of Madrid, Madrid, Spain; 2 Department of Biomedical Sciences and Health Specialties, Care Research Group (GRUPAC) University of La Rioja, Logroño, Spain; 3 Intensive Care Unit, Hospital San Pedro, Logroño, Spain; 4 Intensive Care Unit, Hospital La Paz, Madrid, Spain; 5 Rioja Health Service, Logroño, Spain; 6 Department of Anesthesiology and Reanimation, Salamanca University Hospital, Faculty of Medicine, University of Salamanca, Salamanca, Spain; 7 Department of Critical Care, Reina Sofia Hospital, Tudela, Spain; 8 Division of Scientific Research and Innovation in Emergency Medical Service, Department of Emergency Medical Service, Faculty of Nursing and Midwifery, Wroclaw Medical University, Wroclaw, Poland; 9 Rioja Health Service, Department of Internal Medicine, San Pedro Hospital, Logroño, Spain

**Keywords:** adrenaline, intraosseous access, intravenous access, out-of-hospital cardiac arrest, return of spontaneous circulation

## Abstract

**Introduction:**

Early vascular access is a key component of advanced life support in out-of-hospital cardiac arrest (OHCA), particularly in patients with non-shockable rhythms, in whom early adrenaline administration is recommended. However, the comparative clinical impact of intravenous (IV) versus intraosseous (IO) access in this setting remains uncertain. This study aimed to compare IV and IO vascular access in patients with OHCA and non-shockable rhythms and to evaluate their association with time to first adrenaline administration and return of spontaneous circulation (ROSC).

**Methods:**

We conducted a retrospective observational study including patients with OHCA and a non-shockable initial rhythm attended by Emergency Medical Service 061 La Rioja between January 1, 2022, and December 31, 2024, using data from the Out-of-Hospital Spanish Cardiac Arrest Registry (OHSCAR). Patients in whom cardiopulmonary resuscitation was initiated were eligible. Demographic, temporal, and clinical variables were analyzed. Vascular access was classified as IV or IO. Associations between vascular access route, time to first adrenaline administration, and ROSC status were explored using the chi-square test or Fisher’s exact test, as appropriate.

**Results:**

A total of 185 patients were included. IV access was established in 72.4% of cases (*N* = 134), whereas IO access was used in 27.6% (*N* = 51). Any ROSC was achieved in 33.5% of patients (*N* = 62), and sustained ROSC to hospital arrival was observed in 23.2% (*N* = 43). Initial rhythm was significantly associated with ROSC status (*p* = 0.003). Vascular access route was also significantly associated with ROSC status (*p* = 0.036). Sustained ROSC to hospital arrival was observed in 24.6% of patients with IV access and 19.6% of those with IO access. Earlier adrenaline administration was more frequently observed in the IV group.

**Discussion:**

In this retrospective observational cohort of patients with OHCA and non-shockable rhythms, IV access was associated with a higher proportion of sustained ROSC at hospital arrival compared with IO access. However, this association should be interpreted with caution, as the study design precludes causal inference and residual confounding is likely. In particular, patients receiving IO access may have represented more complex cases, such as those with difficult vascular access or longer resuscitation times.

## 1 Introduction

Out-of-hospital cardiac arrest (OHCA) is the third leading cause of death in Europe (1). Approximately 350,000 OHCAs occur annually across the continent (2). In Europe, the incidence of OHCA in which cardiopulmonary resuscitation (CPR) is initiated is 55 cases per 100,000 inhabitants (3). Only 30% of OHCAs occur in public settings (3). In Spain, the incidence of OHCA in which resuscitation attempts were made was 24.2 cases per 100,000 inhabitants in 2022 (2).

With regard to the cardiac rhythm identified on arrival of emergency medical teams, approximately 20% of OHCAs in Europe present with a shockable rhythm, namely ventricular fibrillation (VF) or pulseless ventricular tachycardia (pVT) (1, 3). In Spain, this proportion reached 23% in 2022 (2). Rhythm identification is crucial, as it determines the therapeutic interventions to be undertaken (3–5).

In any situation suggestive of cardiac arrest, when a person is unresponsive and not breathing or not breathing normally, emergency medical services should be activated by calling 112 or 061 (3, 6, 7). High-quality CPR should then be initiated until an automated external defibrillator (AED) becomes available (3, 6). The AED will analyze the patient’s cardiac rhythm and indicate whether a shock is advised (3, 6). Basic life support (BLS) measures should be continued until the arrival of emergency medical teams, who will then initiate advanced life support (ALS) interventions (8).

Therapeutic management according to the advanced CPR algorithm is determined by the presence or absence of a shockable rhythm (3–6, 9). Rhythm assessment should be performed while minimizing interruptions in chest compressions and, whenever possible, while simultaneously establishing monitoring, optimizing airway and ventilation, and securing vascular access—either intravenous (IV) or intraosseous (IO)—for drug administration (3–6, 9). Identification of a shockable rhythm indicates that defibrillation should be delivered as early as possible. If a normal rhythm is not restored, chest compressions should be resumed immediately after the shock for 2 min, and 1 mg of adrenaline should be administered every 3–5 min via the IV or IO route (3–6, 9).

Conversely, if the patient presents with a non-shockable rhythm, emergency teams should continue chest compressions and administer adrenaline intravenously or intraosseously as early as possible (3–6, 9), regardless of patient age (4, 9–14). The IO route is considered an alternative to IV access (3–6, 9–14). Intraosseous vascular access is simple and rapid to establish and is associated with few complications (8, 15–24). It allows administration of the same drugs at the same doses as the intravenous route, with similar pharmacokinetic and pharmacodynamic properties (8, 15, 17–20, 23, 25). Particularly noteworthy is the speed with which drugs reach the systemic circulation after IO infusion (25–27), reaching the superior vena cava in as little as 2.42 s when the humeral head is used as the insertion site (23).

The aim of the present study was to compare intravenous (IV) and intraosseous (IO) vascular access in patients with out-of-hospital cardiac arrest (OHCA) presenting with a non-shockable rhythm, and to evaluate their association with adrenaline administration time and return of spontaneous circulation (ROSC).

## 2 Methods

This was a retrospective longitudinal observational study conducted over the period 2022–2024. From January 1, 2022, to December 31, 2024, five Class C emergency medical resources (28)—four Advanced Life Support Units (ALSUs) and one helicopter emergency medical service, “Galeno” (HEMS)—belonging to the Emergency Medical Service (EMS) 061 La Rioja (29), provided coverage to the population of La Rioja.

The inclusion criteria comprised all patients with out-of-hospital cardiac arrest whom the Out-of-Hospital Spanish Cardiac Arrest Registry (OHSCAR) form had been completed (2, 30–33) and in whom cardiopulmonary resuscitation (CPR) had been initiated. Patients in whom CPR was not indicated were excluded. Ultimately, only patients presenting with a non-shockable rhythm on EMS arrival were included.

**Figure 1:**
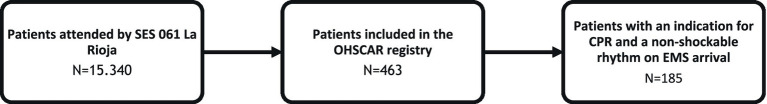
Flow chart.

Figure 1 presents the flowchart summarizing the patient selection process. A total of 15,340 patients were attended by the Class C EMS resources of SES 061 La Rioja during the study period. Overall, 463 OHSCAR records were completed, of which 185 met the inclusion criteria for the present study, corresponding to cases of out-of-hospital cardiac arrest with a non-shockable rhythm in whom CPR was indicated.

Patients who received initial medical care from EMS teams belonging to neighboring Autonomous Communities—Álava, Navarre, and Castile and León—were excluded, as were patients in whom neither peripheral intravenous nor intraosseous vascular access was established by SES La Rioja personnel (*N =* 0 in the latter case).

The study protocol was developed in accordance with the Declaration of Helsinki and approved by the Ethics Committee of the University of La Rioja, with verification code nOjsSvXLLNRvnfVfPr299EBR2092SU2L.

### 2.1 Study variable and definitions

Data collection for the study was performed using the information recorded by healthcare professionals in the Out-of-Hospital Spanish Cardiac Arrest Registry (OHSCAR) (30), in which SES 061 La Rioja participates.

According to the Spanish National Statistics Institute (INE), the mean population of La Rioja during the study period was 322,027 inhabitants (34). In January 2022, the Class C vehicles of SES 061 La Rioja included three Advanced Life Support Units (ALSUs), which provided 24-h coverage to the population of La Rioja, 365 days a year, and were located in Calahorra (Lower Rioja), Haro (Upper Rioja), and Logroño (Central Rioja), identified as ALSU 2, ALSU 3, and ALSU 1, respectively. In April 2023, an additional ALSU began operating in Logroño from 9:00 a.m. to 9:00 p.m. (ALSU 4), and in August the HEMS “Galeno” entered into service (29, 35–37).

The involvement of the entire service team enabled data collection for this registry. Subsequently, one investigator was responsible for checking and validating the recorded information, coding it through a double-system procedure, thereby ensuring the privacy of the study population. The variables included demographic data: age and sex; temporal data: month, year, and time of cardiac arrest (time slot), time of ALS initiation by the EMS, time of first adrenaline administration (categorial variable), and time of termination of CPR maneuvers; as well as clinical data: initial cardiac rhythm, type of vascular access established (intravenous or intraosseous), reason for cannulation, and site in cases of intraosseous vascular access, adrenaline administration, and ROSC.

### 2.2 Statistical analysis

The statistical analysis was performed using IBM SPSS Statistics, version 28.0 (IBM Corp., Armonk, NY, USA). Categorical variables were summarized as absolute frequencies and percentages. Continuous variables were expressed as mean ± standard deviation or median and interquartile range, as appropriate. Normality was assessed using the Kolmogorov–Smirnov test. Associations between categorical variables were analyzed using the chi-square test or Fisher’s exact test, as appropriate. Additionally, a stratified analysis was conducted via contingency tables to assess the association between the primary variables while adjusting for a third variable as a control. A two-sided *p* value < 0.05 was considered statistically significant. Multivariable logistic regression was explored; however, due to the limited number of sustained ROSC events, adjusted models were unstable and at high risk of overfitting. Therefore, the analysis was considered descriptive and exploratory. Missing or unknown data were not imputed.

## 3 Results

From January 1, 2022, to December 31, 2024, the Class C units of SES 061 La Rioja attended a total of 15,340 patients, of whom 463 were diagnosed with out-of-hospital cardiac arrest Cardiopulmonary resuscitation was initiated and deemed indicated in 185 cases. Among these patients, a non-shockable initial rhythm was documented in 26.5% (*N =* 49) in 2022, 37.3% (*N =* 69) in 2023, and 36.2% (*N =* 67) in 2024. Regarding sex distribution, 75.1% (*N =* 139) of patients were male and 24.9% (*N =* 46) were female. Most patients (66.0%, *N =* 122) were aged 50–79 years, whereas 5.4% (*N =* 10) were younger than 20 years. Patients older than 80 years accounted for 15.7% (*N =* 29) of the study population. The distribution of cases by age, sex, and study year is summarized in Table 1.

**Table 1:** Distribution of patients according to age, sex, and year.

Sex	Year																
	2022					2023					2024					Total 22–23–24	
	♂		♀		Total 22	♂		♀		Total 23	♂		♀		Total 24		
Age	*N*	%	*N*	%	*N*	*N*	%	*N*	%	*N*	*N*	%	*N*	%	*N*	*N*	%
0–9	2	50	2	50	4	1	50	1	50	2	1	50	1	50	2	8	4.3
10–19	1	50	1	50	2	0	–	0	–	0	0	–	0	–	0	2	1.1
20–29	0	–	1	100	1	1	100	0	–	1	1	100	0	–	1	3	1.6
30–39	1	100	0	--	1	1	100	0	–	1	1	100	0	–	1	3	1.6
40–49	2	66.7	1	33.3	3	6	66.7	3	33.3	9	6	100	0	–	6	18	9.7
50–59	8	72.7	3	27.3	11	7	87.5	1	12.5	8	10	83.3	2	16.7	12	31	16.8
60–69	6	60	4	40	10	15	83.3	3	16.7	18	16	72.7	6	27.3	22	50	27.0
70–79	7	63.7	4	36.3	11	14	87.5	2	12.5	16	11	78.6	3	21.4	14	41	22.2
80–89	4	66.7	2	33.3	6	7	58.3	5	41.7	12	8	100	0	–	8	26	14.1
90–99	0	–	0	–	0	1	50	1	50	2	1	100	0	–	1	3	1.6
>99	0	–	0	–	0	0	–	0	–	0	0	–	0	–	0	0	0
TOTAL	31	63.3	18	36.7	49	53	76.8	16	23.2	69	55	82.1	12	17.9	67	185	100

*N* represents the absolute frequencies (number of cases) and % represents the relative frequencies (percentage) by sex within each age group. The symbols ♂ and ♀ denote male and female, respectively. The symbol ‘**–**’ indicates that there are no cases in that category.

Analysis of ROSC by sex showed that 28.3% of women achieved return of spontaneous circulation and arrived at the hospital with a pulse, compared with 21.6% of men. Detailed ROSC data by sex are presented in Table 2.

**Table 2:** Association between sex and return of spontaneous circulation.

Sex	NO ROSC		ROSC achieved, but patient died		ROSC achieved		Total		Chi-square test (*p*-value)
	*N*	%	*N*	%	*N*	%	*N*	%	
♂	96	69.0	13	9.4	30	21.6	139	75.1	*p* = 0.432
♀	27	58.7	6	13.0	13	28.3	46	24.9	
Total	123	66.5	19	10.3	43	23.2	185	100.0	

NO ROSC: Return of Spontaneous Circulation was not achieved. ROSC achieved, but patient died: Return of Spontaneous Circulation was achieved, but the patient died before arriving to the hospital. ROSC achieved: Return of Spontaneous Circulation and transported to the hospital with spontaneous circulation. Absolute frequencies (*N*) and relative frequencies (%) reflecting the percentage success (ROSC achieved) or failure (no ROSC or ROSC achieved but patient died) rates within each population group ♂: male and ♀: female.

Most cardiac arrests occurred during the daytime period. Asystole was the most frequent initial rhythm, observed in 81.9% of patients (*N =* 151), followed by pulseless electrical activity in 14.6% (*N =* 27). Overall, ROSC was achieved in 33.5% of patients (*N =* 62), and sustained ROSC to hospital arrival occurred in 23.2% (*N =* 43). Initial rhythm was significantly associated with ROSC status (*p* = 0.003).

Table 3 summarizes the relationship between the patient’s initial rhythm and ROSC.

**Table 3:** Association between initial rhythm and return of spontaneous circulation.

Initial rhythm	NO ROSC		ROSC achieved		ROSC achieved. But patient died		Total		Fisher’s exact test
	*N*	%	*N*	%	*N*	%	*N*	%	
Asystole	108	71.5	30	19.9	13	8.6	151	81.6	*p* = 0.003
Pulseless electrical activity (PEA)	14	51.9	8	29.6	5	18.5	27	14.6	
Non-shockable rhythm identified by AED	0	–	4	80.0	1	20.0	5	2.7	
Unknown	1	50.0	1	50.0	0	–	2	1.1	
Total	123	67.0	43	22.7	19	10.3	185	100	

NO ROSC, Return of Spontaneous Circulation was not achieved. ROSC achieved, but patient died: Return of Spontaneous Circulation was achieved, but the patient died before arriving to the hospital. ROSC achieved: Return of Spontaneous Circulation and transported to the hospital with spontaneous circulation. PEA, Pulseless Electrical Activity; AED, Automated External Defibrillator. Absolute frequencies (*N*) and relative frequencies (%) reflecting the percentage success (ROSC achieved) or failure (no ROSC or ROSC achieved but patient died) rates of the cardiopulmonary resuscitation in relation to the patient’s initial heart rhythm.

Vascular access established for hemodynamic support through the administration of fluids and vasoactive drugs was intravenous (IV) in 72.4% of patients (*N =* 134) and intraosseous (IO) in 27.6% (*N =* 51). Overall, ROSC was achieved in 33.5% of patients (*N =* 62). Among these patients, vascular access was IV in 82.3% (*N =* 51) and IO in 17.7% (*N =* 11). Of the patients who achieved ROSC, 69.4% (*N =* 43) arrived at the hospital with a pulse; of these, 76.7% (*N =* 33) had IV access and 23.3% (*N =* 10) had IO access. A statistically significant association was observed between ROSC and the route of vascular access used (*p* = 0.036), as shown in Table 4. The proportion of patients who achieved ROSC and arrived at the hospital with a pulse was 19.6% (*N =* 10) among those with IO access, compared with 24.6% (*N =* 33) among those with IV access. The association between adrenaline administration time and route of administration is presented in Table 5.

**Table 4:** Association between drug administration route and return of spontaneous circulation.

Route	NO ROSC		ROSC achieved		ROSC achieved, but patient died		Total		Fisher’s exact test
	*N*	%	*N*	%	*N*	%	*N*	%	
IV	83	67.5	33	76.7	18	94.7	134	72.4	*P* = 0.036
IO	40	32.5	10	23.3	1	5.3	51	27.6	
Total	123	66.5	43	23.2	19	10.3	185	100	

IV, intravenous access; IO, intraosseous vascular access; NO ROSC, Return of Spontaneous Circulation was not achieved. ROSC achieved, but patient died: Return of Spontaneous Circulation was achieved, but the patient died before arriving to the hospital. ROSC achieved: Return of Spontaneous Circulation and transported to the hospital with spontaneous circulation. Absolute frequencies (N) and relative frequencies (%) reflecting the percentage success (ROSC achieved) or failure (no ROSC or ROSC achieved but patient died) rates with regard to the vascular access used in cardiopulmonary resuscitation.

**Table 5:** Association between adrenaline administration time and route of administration.

Route	<2		>2 < 3:59		>4 < 5:59		>6 < 7:59		>8 < 9:59		>10		UNK		NO APP		Total	
	*N*	%	*N*	%	*N*	%	*N*	%	*N*	%	*N*	%	*N*	%	*N*	%	*N*	%
IV	20	95.2	38	70.4	28	73.7	16	64.0	9	52.9	19	95.0	0	–	4	66.7	134	72.4
IO	1	4.8	16	29.6	10	26.3	9	36.0	8	47.1	1	5.0	4	100.0	2	33.3	51	27.6
Total	21	100.0	54	100.0	38	100.0	25	100.0	17	100.0	20	100.0	4	100.0	6	100.0	185	100.0

IV, intravenous access; IO, intraosseous access; UNK, unknown; NO APP, not applicable. Time to first adrenaline administration was analyzed using predefined registry intervals. Differences between IV and IO groups were assessed using the chi-square test or Fisher’s exact test, as appropriate. Percentages refer to the distribution of vascular access route within each adrenaline timing interval”.

Given the limited number of sustained ROSC events, multivariable adjusted results were not retained as confirmatory analyses.

## 4 Discussion

Cases in which advanced life support (ALS) was initiated in patients with an indicated non-shockable rhythm represented 39.9% (*N =* 185). The EuReCa TWO study on out-of-hospital cardiac arrest (OHCA) survival in Europe, published in 2020, reported that 20% of initial rhythms identified by healthcare professionals were shockable (1), a finding consistent with the epidemiological data presented in the 2025 European Resuscitation Council Guidelines (3).

Regarding age and sex among patients with cardiac arrest in whom ALS was indicated and the initial rhythm was non-shockable, a clear male predominance was observed, reaching a ratio of up to 3:1 according to several authors (2, 32, 38), except in younger age groups, where the distribution between sexes was more balanced. These findings suggest that sex alone is not a determinant of return of spontaneous circulation (ROSC). In this regard, Cruz-Garcinuño et al. (39), in a retrospective observational study published in September 2025, highlighted the need to analyze sex as a dependent variable because of its interaction with other relevant factors, including age, comorbidities, bystander cardiopulmonary resuscitation (CPR), and access to treatment. Similarly, initial rhythm, arrest location, and patient age have also been shown to influence ROSC (40). Overall, these data suggest that sex-related differences in ROSC are likely multifactorial rather than attributable to sex alone (41, 42). Indeed, some studies have reported a trend toward higher ROSC rates in men, possibly because they are more likely to present with ventricular fibrillation (VF) or pulseless ventricular tachycardia (pVT) (40).

The findings of the present study regarding ROSC according to initial rhythm are also noteworthy. Overall, 23.2% of patients (*N =* 43) regained a pulse and maintained it until hospital arrival. This proportion is consistent with previous reports describing ROSC rates after OHCA between 25 and 30%, including both shockable and non-shockable rhythms (1, 2, 32, 38, 43). Among patients presenting with asystole, who accounted for 81.6% of cases, ROSC was achieved in only 19.9%. In contrast, among those whose initial rhythm was pulseless electrical activity (PEA), representing 14.6% of cases (*N =* 27), the success rate of resuscitation maneuvers was 29.6% (*N =* 8). Notably, among patients in whom a non-shockable rhythm was identified by an automated external defibrillator (AED) before emergency medical services (EMS) arrival, ROSC was achieved in 100% of cases, although only 80% arrived at hospital with a pulse. This finding reinforces the importance of early resuscitation efforts initiated by bystanders and the availability of public-access defibrillation in improving cardiac arrest survival (1–3, 6). Collectively, these results underscore the need to strengthen public education on cardiac arrest recognition, activation of emergency response systems, and early initiation of CPR. Introducing such training at an early age is particularly relevant and further supports the importance of the “Kids Save Lives” initiative as a public health priority (3, 38, 44, 45).

In this study, intravenous (IV) vascular access, which was associated with a higher ROSC rate (24.6%), was also associated with ROSC status in the unadjusted analysis of adrenaline administration during OHCA management when compared with intraosseous (IO) access, in line with previous literature (3, 14, 15, 46–51). The data also showed that the choice of vascular access had a substantial impact on the timing of adrenaline administration. IV access was associated both with the shortest time to drug delivery (<2 min) and, paradoxically, with the longest delay (>10 min). This pattern suggests that IV access should remain the first-choice route when it can be established promptly. However, when IV cannulation is difficult or not immediately feasible, IO access should not be unnecessarily delayed, given its ease of insertion, rapid deployment, and high first-attempt success rate reported in previous studies (8, 15, 17, 21, 48, 49). This is particularly relevant in patients with non-shockable rhythms, in whom early adrenaline administration is recommended (3, 14, 52).

One possible explanation for delayed adrenaline administration through the IO route is that IO access may have been established only after failed IV attempts or after excessive time had been spent recognizing the difficulty of IV cannulation (52). This delay may, in turn, have contributed to the lower ROSC rates observed with IO access. In this context, the cohort analysis by Rosell et al. (38) suggested that the first dose of epinephrine administered through IO access was associated with a longer interval to ROSC compared with IV access (13.2 min vs. 14.9 min for tibial IO; lower hazard ratio for IO). These findings suggest that, although IO access provides a rapid alternative route for vascular access, any procedural time advantage does not necessarily translate into improved clinical outcomes. Furthermore, the same study found that each additional minute of delay in adrenaline administration after EMS arrival was associated with a significant reduction in hospital survival (38).

Nevertheless, this association should be interpreted cautiously, as multivariable adjustment was limited by the small number of outcome events and the risk of overfitting. Therefore, the findings should be regarded as exploratory and hypothesis-generating.

## 5 Limitations

This study has several limitations and strengths. A major limitation is the relatively small population of the Autonomous Community of La Rioja, which comprises approximately 322,000 inhabitants. However, the incidence of cardiac arrest per 100,000 population is comparable to that reported in large European registries such as EuReCa One and EuReCa two. These findings should be considered preliminary and hypothesis-generating. Larger multicenter studies, ideally with standardized data collection and adjustment for relevant confounders, are needed to determine whether vascular access route independently influences outcomes in OHCA with non-shockable rhythms.”

A limitation of this study is its retrospective observational design, which precludes causal inference. In particular, IO access may have been selected for patients with more difficult vascular access, delayed access establishment, longer resuscitation duration, or poorer clinical condition. Therefore, residual confounding—especially confounding by indication—cannot be excluded, and the observed associations should not be interpreted as evidence of a cause-and-effect relationship. A robust multivariable analysis could not be performed due to the limited number of sustained ROSC events and the risk of overfitting.

A key strength of the study is the strong commitment and engagement of the SES 061 La Rioja staff, which enabled the implementation of an out-of-hospital cardiac arrest registry. The establishment of this registry represents an essential step not only for clinical research, but also for the continuous improvement of care quality and the overall management of OHCA, ultimately contributing to better patient outcomes.

Some subgroup analyses were based on very small numbers, which limits the precision and robustness of the observed associations.

Moreover, the present study may provide a useful foundation for future research with longer follow-up periods and larger datasets.

## 6 Conclusion

IV access was associated with a higher proportion of sustained ROSC in this cohort during advanced life support (ALS) for out-of-hospital cardiac arrest (OHCA). However, these findings should be interpreted as exploratory and hypothesis-generating, as the observational design and lack of robust multivariable adjustment preclude causal inference. Nevertheless, when IV access cannot be established promptly, intraosseous (IO) access should be initiated without unnecessary delay, particularly in patients with non-shockable rhythms, in whom early adrenaline administration is a key determinant of return of spontaneous circulation (ROSC). Accordingly, healthcare professionals should be adequately trained not only in the technical aspects of IV and IO access, but also in the critical decision-making required to determine when further attempts at one route are unlikely to be beneficial and when prompt transition to an alternative route is warranted.

## Data Availability

The raw data supporting the conclusions of this article will be made available by the first author upon reasonable request, in accordance with personal data protection regulations.

## References

[1] Gräsner JT Wnent J Herlitz J Perkins GD Lefering R Tjelmeland I . Survival after out-of-hospital cardiac arrest in Europe - results of the EuReCa TWO study. Resuscitation. (2020) 148:218–26. doi: 10.1016/j.resuscitation.2019.12.042 32027980

[2] Ruiz Azpiazu JI Fernández Del Valle P Carmen Escriche M Royo Embid S Fernández Barreras C Azeli Y . Incidencia, tratamiento y factores asociados con la supervivencia de la parada cardiaca extrahospitalaria atendida por los servicios de emergencias en España: informe 2022 del registro OHSCAR. Emergencias Rev la Soc Esp Med Emergencias. (2024) 36:131–9. 10.55633/s3me/014.2024 38597620

[3] Greif R Lauridsen KG Djärv T Ek JE Monnelly V Monsieurs KG . European Resuscitation Council Guidelines 2025 Executive Summary. Resuscitation. (2025) 215:110770. doi: 10.1016/j.resuscitation.2025.110770 41117573

[4] Soar J Böttiger BW Carli P Jiménez FC Cimpoesu D Cole G . European Resuscitation Council Guidelines 2025 Adult Advanced Life Support. Resuscitation. (2025) 215:110769. doi: 10.1016/j.resuscitation.2025.110769 41117572

[5] Wigginton JG Agarwal S Bartos JA Coute RA Drennan IR Haamid A . Part 9: Adult Advanced Life Support: 2025 American Heart Association Guidelines for Cardiopulmonary Resuscitation and Emergency Cardiovascular Care. Circulation. (2025) 152:S538–77. doi: 10.1161/CIR.0000000000001376 41122884

[6] Rios M Del Bartos JA Panchal AR Atkins DL Drennan IR Elmer J (2025). Part 1: executive summary: 2025 American Heart Association guidelines for cardiopulmonary resuscitation and emergency cardiovascular care. 10.1161/CIR.0000000000001372 41122893

[7] Ministry of Public Works. Royal Decree 903/1997, of 16 June, regulating access, through telecommunications networks, to the emergency call service via the telephone number 112. Official State Gazette. (1997) 153:19953–5. Available online at: https://www.boe.es/eli/es/rd/1997/06/16/903

[8] Burgos-Esteban A Quintana-Diaz M Cordón-Hurtado V Giménez-Luzuriaga M Santolalla-Arnedo I de Viñaspre-Hernánz RR . Epidemiology, use, and practice of the intraosseous route in an out-of-hospital emergency department: a retrospective cross-sectional study. Front Public Health. (2024) 12, 2:1–7. doi: 10.3389/fpubh.2024.1375431 PMC11061842 38694974

[9] Drennan IR Berg KM Böttiger BW Chia YW Couper K Crowley C . Advanced Life Support Task Force Collaborators. Advanced Life Support: 2025 International Liaison Committee on Resuscitation Consensus on Science With Treatment Recommendations. Circulation. (2025) 152:S72–S115. doi: 10.1161/CIR.0000000000001360 41122842

[10] Liley HG Weiner GM Wyckoff MH Rabi Y Schmölzer GM Almeida MF De . Neonatal Life Support: 2025 International Liaison Committee on Resuscitation Consensus on Science With Treatment Recommendations. Pediatrics. (2026) 157:e2025074766. doi: 10.1542/peds.2025-074766 41122837

[11] Scholefield BR Acworth J Ng K Tiwari LK Raymond TT Christoff A . Pediatric life support: 2025 International Liaison Committee on Resuscitation consensus on science with treatment recommendations. Circulation. (2025) 152. doi: 10.1161/CIR.0000000000001362 41122843

[12] Hogeveen M Monnelly V Binkhorst M Cusack J Schwindt E Soleva AL . European resuscitation council guidelines 2025 newborn resuscitation and support of transition. Resuscitation. (2025) 1:110766. doi: 10.1016/j.resuscitation.2025.110766 41117576

[13] Djakow J Mcbeth N Skellett S Buysse CMP Cardona F Lucas N De European Resuscitation Council Guidelines 2025 Paediatric Life Support. Resuscitation. (2025) 1:110767. doi: 10.1016/j.resuscitation.2025.110767 41117571

[14] American Heart Association. Highlights of the 2025 American Heart Association Guidelines for CPR and ECC. American Heart Association. (2025). Available online at: https://cpr.heart.org/-/media/CPR-Files/2025-documents-for-cpr-heart-edits-posting/Resuscitation-Science/JN1582_ESXM_Hghlghts_2025ECCGuidelines_Final_251022.pdf?sc_lang=en

[15] Constante P Gómez V Felipe E Cristóbal J Leal PBC. Manejo de la vía intraósea en situaciones de urgencia. Revisión sistemática. Rev Sanit Investig. (2021) 21:1–12.

[16] Rijnhout TWH Kieft M Klein WM Tan ECTH. Effectiveness of intraosseous access during resuscitation: a retrospective cohort study. BMC Emerg Med. (2024) 24:192. doi: 10.1186/s12873-024-01103-w 39407103 PMC11475784

[17] Domínguez Romero A Ciprés Añaños E. Vía intraósea como alternativa al acceso intravenoso. Rev Sanit Investig. (2020) 1:1–9.

[18] García C Garcés P García M Barcelona C García LMY. Vía intraósea en el paciente crítico. Artículo monográfico. Rev Sanit Investig. (2021) 22:1–9.

[19] Fernández-Utrilla Miguel P López Manzano EM Vega Arjona E. Una alternativa que salva vidas. Vía Intraósea. Paraninfo Digit. (2020) XIV:1–3.

[20] Villena Esteo O. La vía intraósea en situaciones de emergencia: Análisis en el medio extrahospitalario. Emergencias. (2012) 24:44–6.

[21] Martínez García Alcaide S Delgado Diéguez R Herraiz Martínez Y Aviol Oliveros A Bernal Fradejas A Barcelona Tambo T. Utilización de la vía intraósea como acceso venoso. Revisión sistemática [Use of the intraosseous route as venous access. Systematic review]. Rev Sanit Investig. (2021) 12:1–15.

[22] Martínez Tapia A. Comparación de la vía intraósea e intravenosa en la parada cardiorrespiratoria extrahospitalaria ¿Debería la enfermería de emergencias considerar la vía intraósea como primera opción en los pacientes adultos? Rev Científica del CODEM. (2019) 3:29–42.

[23] Vidacare Corporation. The Science & Fundamentals of intraosseous vascular access. San Antonio, TX: Vidacare Corporation (2020).

[24] Leidel BA Kirchhoff C Bogner V Stegmaier J Mutschler W Kanz K-G . Is the intraosseous access route fast and efficacious compared to conventional central venous catheterization in adult patients under resuscitation in the emergency department? A prospective observational pilot study. Patient Saf Surg. (2009) 3:24. doi: 10.1186/1754-9493-3-24, 19814822 PMC2764565

[25] Mas Company E Díaz Gómez M de la Castejón Encina ME. Fármacos y fluidos administrados por vía intraósea en pacientes críticos: revisión sistemática. Rev Española Urgencias y Emergencias. (2022) 1:97–103.

[26] José Antonio Iglesias V María Luisa Chayán Z. Dispositivos de acceso intraóseo: un ensayo clínico efectuado con asignación aleatoria y control para la comparación de 3 dispositivos de acceso intraóseo. Prehosp Emerg Care. (2010) 3:187–97.

[27] Schalk R Schweigkofler U Lotz G Zacharowski K Latasch L Byhahn C. Efficacy of the EZ-IO ® needle driver for out-of- hospital intraosseous access - a preliminary, observational, multicenter study. Scand J Trauma Resusc Emerg Med. (2011). 19:65. doi: 10.1186/1757-7241-19-65 22029625 PMC3212886

[28] Ministerio de la Presidencia, Sistemas de Información de Atención Primaria, Ministerio de Transportes Turismo y Comunicaciones J del E. (2019). Real Decreto 836/2012, de 25 de mayo, por el que se establecen las características técnicas, el equipamiento sanitario y la dotación de personal de los vehículos de transporte sanitario por carretera. Available online at: https://www.sanidad.gob.es/estadEstudios/estadisticas/docs/siap/Definiciones.pdf%0Ahttp://www.cancer.org/cancer/news/news/world-health-organization-says-processed-meat-causes-cancer (accessed March 15, 2026).

[29] Riojasalud. (2026). Emergencias Sanitarias 061 - Sobre el Servicio. Available online at: https://www.riojasalud.es/servicios/emergencias-sanitarias-061 (accessed March 1, 2026).

[30] Registro OHSCAR - CERCP. (2026). Available online at: https://www.cercp.org/registro-ohscar/ (accessed February 1, 2026).

[31] Rosell-Ortiz F Escalada-Roig X Fernández Del Valle P Sánchez-Santos L Navalpotro-Pascual JM Echarri-Sucunza A . Out-of-hospital cardiac arrest (OHCA) attended by mobile emergency teams with a physician on board. Results of the Spanish OHCA Registry (OSHCAR). Resuscitation. (2015) 113:90–95. doi: 10.1016/j.resuscitation.2017.01.029 28202420

[32] Ruiz-Azpiazu JI Daponte-Codina A Del Valle PF López-Cabeza N Jiménez-Fàbrega FX Iglesias-Vázquez JA . Regional variation in the incidence, general characteristics, and outcomes of prehospital cardiac arrest in Spain: the out-of-hospital Spanish cardiac arrest registry. Emergencias. (2021) 33:15–22. 33496395

[33] Ruiz Azpiazu JI Fernández Del Valle P Echarri Sucunza A Iglesias Vázquez JA Del Pozo C Knox ECL . Out-of-hospital cardiac arrest following the COVID-19 pandemic. JAMA Netw Open. (2024) 7:1–14. doi: 10.1001/jamanetworkopen.2023.52377, 38261321 PMC10807256

[34] Población según comunidad autónoma y provincia y sexo (67988) (2026). Available online at: https://www.ine.es/jaxiT3/Datos.htm?t=67988 (accessed March 7, 2026).

[35] de Salud SR (2025). Gonzalo Capellán felicita al servicio 061 en su 25o aniversario “habiéndose ganado la confianza de los riojanos por su eficiencia y capacidad resolutiva” - Rioja Salud. Available online at: https://www.riojasalud.es/institucion/actualidad/2025/02/gonzalo-capellan-felicita-al-servicio-061-en-su-25-aniversario-habiendose-ganado-la-confianza-de-los-riojanos-por-su-eficiencia-y-capacidad-resolutiva (accessed March 15, 2026).

[36] Félez N. (2023). Galeno: el pájaro de hierro que salva vidas. Available online at: https://nuevecuatrouno.com/2023/08/19/galeno-el-pajaro-de-hierro-que-salva-vidas/ (accessed August 19, 2023).

[37] Servicio Riojano de Salud. 2023 Annual Report [Internet]. Logroño: Servicio Riojano de Salud (2023). [cited 2026 Mar 1]. Available from: https://www.riojasalud.es/files/content/institucion/Memoria_SRS_2023.pdf

[38] Rosell Ortiz F García Del Águila J Fernández Del Valle P Mellado-Vergel FJ Vergara-Pérez S Ruiz-Montero MR . Supervivencia y factores asociados a la práctica de reanimación cardiopulmonar en curso entre los pacientes con parada cardiaca extrahospitalaria. Emergencias. (2018) 30:156–62. 29687669

[39] Cruz-Garcinuño M Juárez-Vela R Chover-Sierra E Navas-Echazarreta N Czapla M Ballestar-Tarín ML . Sex differences and gender-oriented characteristics in intensive care unit admissions for patients with traumatic brain injury in Spain. Front Med. (2025) 12:1–12. doi: 10.3389/fmed.2025.1622422, 41080953 PMC12511112

[40] Briz VD Juárez-vela R Lewandowski Ł Kubielas G (2024). Sex-related differences in the association of obesity described by emergency medical teams on outcomes in out-of-hospital cardiac arrest patients. 10.17219/acem/193367 39508211

[41] Lei H Hu J Liu L Xu D. Sex differences in survival after out-of-hospital cardiac arrest: a meta-analysis. Crit Care. (2020) 24:1–13. doi: 10.1186/s13054-020-03331-5, 33076963 PMC7570116

[42] Lakbar I Ippolito M Nassiri A Delamarre L Tadger P Leone M . Sex and out-of-hospital cardiac arrest survival: a systematic review. Ann Intensive Care. (2022) 12:1091. doi: 10.1186/s13613-022-01091-9, 36534195 PMC9763524

[43] Gräsner JT Lefering R Koster RW Masterson S Böttiger BW Herlitz J . EuReCa ONE—27 nations, ONE Europe, ONE registry: a prospective one month analysis of out-of-hospital cardiac arrest outcomes in 27 countries in Europe. Resuscitation. (2016) 105:188–95. doi: 10.1016/j.resuscitation.2016.06.004, 27321577

[44] Abelairas-Gómez C Carballo-Fazanes A Martínez-Isasi S López-García S Rico-Díaz J Rodríguez-Núñez A. Conocimiento y actitudes sobre los primeros auxilios y soporte vital básico de docentes de Educación Infantil y Primaria y los progenitores PALABRAS CLAVE. An Pediatr. (2020) 92:268–76. 10.1016/j.anpedi.2019.10.010 31870834

[45] Dezfulian C Cabañas JG Buckley JR Cash RE Crowe RP Drennan IR . Part 4: systems of care: 2025 American Heart Association guidelines for cardiopulmonary resuscitation and emergency cardiovascular care. New York, NY: American Heart Association (2025). 10.1161/CIR.0000000000001378 PMC13003348 41122886

[46] Rosell-Ortiz F Escalada-Roig X Fernández del Valle P Sánchez-Santos L Navalpotro-Pascual JM Echarri-Sucunza A . Out-of-hospital cardiac arrest (OHCA) attended by mobile emergency teams with a physician on board. Results of the Spanish OHCA registry (OSHCAR). Resuscitation. (2017) 113:90–5. doi: 10.1016/j.resuscitation.2017.01.029, 28202420

[47] Couper K Andersen LW Drennan IR Grunau BE Kudenchuk PJ Lall R . Intraosseous and intravenous vascular access during adult cardiac arrest: a systematic review and meta-analysis. Resuscitation. (2025) 207:1–15. 10.1016/j.resuscitation.2024.110481 39742938

[48] Kokori E Al-hashemi N Aldeen ZS Patel R Aderinto N Olatunji G. Intraosseous vs. Intravenous access in out-of- hospital cardiac arrest: a systematic review and meta-analysis of clinical outcomes. Int J Emerg Med. (2025) 18:927. doi: 10.1186/s12245-025-00927-y, 40665212 PMC12261587

[49] Baert V Vilhelm C Escutnaire J Nave S Hugenschmitt D Chouihed T . Intraosseous versus peripheral intravenous access during out-of-hospital cardiac arrest: a comparison of 30-day survival and neurological outcome in the French National Registry. Cardiovasc Drugs Ther. (2020) 34:189–97. doi: 10.1007/s10557-020-06952-8, 32146637

[50] Nguyen L Suarez S Daniels J Sanchez C Landry K Redfield C. Effect of intravenous versus intraosseous access in prehospital cardiac arrest. Air Med J. (2019) 38:147–9. doi: 10.1016/j.amj.2019.02.005, 31122576

[51] Zhang Y Zhu J Liu Z Gu L Zhang W Zhan H . Intravenous versus intraosseous adrenaline administration in out-of-hospital cardiac arrest: a retrospective cohort study. Resuscitation. (2020) 149:209–16. doi: 10.1016/j.resuscitation.2020.01.009, 31982506

[52] Morales-Cané I Valverde-León M d R Rodríguez-Borrego MA López-Soto PJ. Intraosseous access in adults in cardiac arrest: a systematic review and meta-analysis. Emergencias. (2020) 32:49–56. 31909913

